# A quantitative, Bayesian-informed approach to gene-specific variant classification: Updated Expert Panel recommendations improve classification of *TP53* germline variants for Li-Fraumeni syndrome

**DOI:** 10.1186/s13073-025-01536-3

**Published:** 2025-10-22

**Authors:** Cristina Fortuno, Megan N. Frone, Jessica Mester, Miguel de la Hoya, Phuong L. Mai, Tina Pesaran, Maria Isabel Achatz, Rebecca Bassett, Carolina Bustamante, Stephanie Crowley, Kelvin Cesar de Andrade, D. Gareth Evans, Bingjian Feng, Laura Fuqua, Maria Isabel Harrell, Jessica N. Hatton, Robert Huether, Chimene Kesserwan, Kristy Lee, Suzanne P. MacFarland, Jamie L. Maciaszek, Kara Maxwell, Kelly McGoldrick, Maureen Murphy, Bita Nehoray, Judith Penkert, Emilia Modolo Pinto, Sharon E. Plon, Alison Schwartz-Levine, Ashley S. Thompson, Wenyi Wang, Gerard P. Zambetti, Kristin Zelley, Paul A. James, Sharon A. Savage, Christian P. Kratz, Amanda B. Spurdle

**Affiliations:** 1 Population Health Program, QIMR Berghofer Medical Research Institute, Brisbane, QLD Australia; 2 Clinical Genetics Branch, Division of Cancer Epidemiology and Genetics, National Cancer Institute, National Institutes of Health, Rockville, MD USA; 3 GeneDx, Gaithersburg, MD USA; 4 Molecular Oncology Laboratory, Hospital Clinico San Carlos, Instituto de Investigación Sanitaria San Carlos (IdISSC), Madrid, Spain; 5 Center for Clinical Genetics and Genomics, Department of Obstetrics, Gynecology, and Reproductive Sciences, University of Pittsburgh, Pittsburgh, PA USA; 6 Ambry Genetics, Aliso Viejo, CA USA; 7 Centro de Oncologia, Hospital Sírio-Libanês, São Paulo, Brazil; 8 Moanalua Medical Center, Hawaii Permanente Medical Group, Kaiser Permanente, Honolulu, HI USA; 9 OC Precision Medicine, São Paulo, Brazil; 10 Invitae Corporation, San Francisco, CA USA; 11 Division of Evolution, Infection and Genomics, School of Biological Sciences, Faculty of Biology, Medicine and Health, The University of Manchester, Manchester, UK; 12 University of Utah, Salt Lake City, UT USA; 13 Baylor Genetics, Houston, TX USA; 14 Labcorp Genetics (Formerly Invitae Corp), Burlington, NC USA; 15 Tempus AI, Chicago, IL USA; 16 Department of Pathology, NYU Grossman School of Medicine, New York, NY USA; 17 Department of Genetics, University of North Carolina at Chapel Hill, Chapel Hill, NC USA; 18 Division of Oncology, Children’s Hospital of Philadelphia, Philadelphia, PA USA; 19 Department of Pathology, St. Jude Children’s Research Hospital, Memphis, TN USA; 20 Program in Molecular and Cellular Oncogenesis, The Wistar Institute, Philadelphia, USA; 21 Division of Clinical Cancer Genomics, Departments of Medical Oncology and Therapeutics Research and Population Sciences, City of Hope, Duarte, CA USA; 22 Department of Human Genetics, Hannover Medical School, Hannover, Germany; 23 Pediatric Hematology and Oncology, Hannover Medical School, Hannover, Germany; 24 Dan L. Duncan Cancer Center and the Department of Pediatrics, Baylor College of Medicine, Houston, TX USA; 25 Division of Cancer Genetics and Prevention, Dana-Farber Cancer Institute, Boston, MA USA; 26 Department of Pathology, Microbiology, and Immunology, Molecular Diagnostics Section, Vanderbilt Medical Laboratories, Nashville, TN USA; 27 Department of Bioinformatics and Computational Biology, The University of Texas MD Anderson Cancer Center, Houston, TX USA; 28 Familial Cancer Centre, Peter MacCallum Cancer Centre, Melbourne, VIC Australia

**Keywords:** *TP53*, Li-Fraumeni syndrome, ACMG guidelines, ClinGen Variant Curation Expert Panel (VCEP)

## Abstract

**Background:**

Germline pathogenic variants in *TP53* cause Li-Fraumeni syndrome, with significantly elevated cancer risk from infancy. Accurate classification of *TP53* variants is essential to guide clinical management and surveillance, yet many variants remain classified as variants of uncertain significance (VUS). To improve classification accuracy and reduce the proportion of VUS, the ClinGen *TP53* Variant Curation Expert Panel (VCEP) has updated its specifications.

**Methods:**

The updated specifications incorporate the latest ClinGen recommendations and methodological advances, providing greater granularity for multiple evidence types, and also introduce the novel use of variant allele fraction as evidence of pathogenicity, particularly in the context of clonal hematopoiesis. Whenever feasible, the VCEP followed a data-driven approach using likelihood ratio-based quantitative analyses to guide code application and determine strength modifications, while also factoring in expert judgment. Proposed modifications were first discussed in working group meetings and then subjected to comprehensive review during monthly general VCEP meetings to reach consensus.

**Results:**

The performance of new specifications was compared to that of the old specifications for 43 pilot variants, and led to both decreased VUS and increased certainty, with clinically meaningful classifications for 93% of variants.

**Conclusions:**

The updated *TP53* specfications are expected to reduce VUS rates, increase inter-laboratory concordance, and improve medical management for individuals with germline *TP53* variants. The most current version is available at the ClinGen Criteria Specifications Registry (CSpec): https://cspec.genome.network/cspec/ui/svi/svi/GN009.

**Supplementary Information:**

The online version contains supplementary material available at 10.1186/s13073-025-01536-3.

## Background

Germline variants in *TP53*, a tumor suppressor gene responsible for maintaining genome stability, are the only known cause of Li-Fraumeni syndrome (LFS) (MONDO:0018875) [1]. LFS is characterized by an increased risk of developing many cancer types, usually manifesting at an early age [2, 3]. The most characteristic LFS-associated cancers are premenopausal breast cancer, sarcoma, adrenocortical carcinoma, and brain tumors; however, the cancer spectrum is known to be much wider [4–6].

Given the high cancer penetrance associated with pathogenic/likely pathogenic (P/LP) germline *TP53* variants, their misclassification can have severe consequences, with false positives potentially leading to unnecessary interventions and harm from overscreening and false negatives potentially leading to missed opportunities to reduce cancer-related morbidity and mortality [7, 8]. Both concerns apply to patients with variants of uncertain significance (VUS), in addition to increased patient anxiety [9].

The American College of Medical Genetics and Genomics and the Association for Molecular Pathology (ACMG/AMP) has had a pivotal role in developing global standards for germline variant classification [10]. Clinical Genome Resource (ClinGen) Variant Curation Expert Panels (VCEPs) are collaborative groups that bring together experts in phenotype, molecular diagnosis, and gene function with the goal of improving and applying gene-specific specifications to particular genes [11]. Variant Curation Expert Panels within this National Institutes of Health (NIH)-funded resource follow the Food and Drug Administration (FDA)-recognized guidance for Public Human Genetic Variant databases and the ClinGen VCEP process to develop and pilot gene-specific modifications of the ACMG/AMP variant curation guidelines. To mitigate the challenges of accurate *TP53* variant classification, the ClinGen *TP53* VCEP [12] formed in 2015 and released the first *TP53*-specific classification guidelines (v1) to the ClinGen *TP53* VCEP website in 2019 [13], which has since undergone several minor revisions (available in the Criteria Specification Registry (CSpec)). Applying our *TP53* v1 specifications to a set of 43 pilot variants demonstrated high intra-biocurator consistency, reduced clinically relevant discrepancies (i.e., between pathogenic and benign), and resolved VUS classifications into pathogenic or benign classifications. Since the publication of the initial specifications, the *TP53* VCEP has curated 119 *TP53* variants, which have been deposited in the ClinVar database as three-star submissions [14]. Additional granular curation details for the variants are also available at the ClinGen Evidence repository (ERepo) [15].

Given the continuously updated recommendations from the ClinGen Sequence Variant Interpretation (SVI) working group [16–18], new evidence types and tools becoming available for variant classification [19–21], and ongoing feedback from biocurators actively using these specifications, it became clear that updates to the *TP53* specifications were needed to facilitate resolution of more VUS and lead to an overall improvement in *TP53* variant curation and classification. This manuscript outlines the efforts of the ClinGen *TP53* VCEP in developing such specifications (v2) and presents the outcomes obtained from pilot variants.

## Methods

### ClinGen *TP53* VCEP approach to criteria modifications

The group that developed the updated *TP53* specifications consisted of 37 individuals, including two co-chairs and one coordinator, with representation from expert clinicians, genetic counselors, research and laboratory scientists, variant scientists, and clinical laboratory molecular directors. Group members were from 28 institutions in five countries (Australia, Brazil, Germany, Spain, and the USA). The VCEP members were divided into three working subgroups: Population/Computational, Functional, and Phenotype. During the period of active rules revision, each subgroup met monthly to discuss ACMG/AMP criteria updates pertaining to their focus, in parallel with regular email communications.

Whenever feasible, the VCEP followed a data-driven approach using likelihood ratio (LR)-based quantitative analyses to guide code application and corresponding strengths, while also factoring in expert judgment, applying previously used LR estimation methods [22] that have been adopted by other VCEPs [23]. This included analyses previously done as part of independent studies [19, 20, 24] as well as new analyses specifically done in the context of VCEP activities, as explained below. The VCEP considered any new evidence available with potential to be included in *TP53* germline variant classification, and new recommendations directly arising from the ClinGen SVI and/or those supported by the SVI.

All proposed modifications, including criteria combinations to achieve classifications, were discussed among the corresponding members during working group meetings. These proposals were then further subjected to a comprehensive review during additional monthly general VCEP meetings to reach consensus.

The modified updated specifications were submitted to the SVI for review and, after consideration of feedback and subsequent modification, were approved as v2.0 on 7/24/2024, which were then released to the Criteria Specification Registry (CSpec). Since approval, there have been minor updates to the specifications clarifying language for ease of use but not changing the substance of the rules, with the current version as of publication being v2.3.0.

### Application of updated *TP53* specifications to pilot variants

The updated criteria were piloted on the original 43 v1.0 *TP53* pilot variants to assess performance. Pilot variants were chosen to cover varying molecular effects and to adequately test the usability of updated codes while ensuring good performance across a spectrum of benign and pathogenic variants. The latest ClinVar (as of 1 March 2024) ClinGen *TP53* VCEP classification was used for comparison. These pilot variants included 14 variants previously classified by the VCEP as Pathogenic (P), nine as Likely Pathogenic (LP), four as VUS, ten as Likely Benign (LB), and six as Benign (B). Seven biocurators re-curated the pilot variants with the v2 specifications utilizing the ClinGen Variant Curation Interface [14]. In addition to publicly curated data from the literature and the NIH-hosted *TP53* Database [25], unpublished de-identified clinical data were obtained from VCEP members, including information from College of American Pathologists-accredited and Clinical Laboratory Improvement Amendments-certified clinical diagnostic laboratories and the National Cancer Institute Li-Fraumeni syndrome study (ClinicalTrials.gov NCT01443468). Biocurators reviewed curated variants on a monthly Biocurator call. Variants were also preliminarily reviewed by the Biocurator Trainers who additionally serve as Core Approvers. Variants were curated using ClinGen VCEP and Variant Curation Standard Operating Procedures [26]. All variants were reviewed and approved by the VCEP members and at least three Core Approver members. Pilot variant classifications using the *TP53* v2 specifications are publicly available in both ClinVar and the ERepo.

## Results

In the v1 specifications of the *TP53* specifications, nine (PM3, PM4, PP2, PP4, PP5, BP1, BP3, BP5, BP6) codes were excluded and 19 of the 28 original ACMG/AMP criteria were modified. In the *TP53* VCEP’s specifications v2, all criteria were reassessed and underwent modifications, including major and minor revisions. Two additional criteria are now excluded as part of the revisions (PM6 and BP2), and a previously excluded criterion (PP4) has been reintroduced to capture a new evidence type. Consequently, the updated *TP53* specifications use 18 criteria (Table 1). Table 1 highlights the main differences of the *TP53* specifications v2 compared to v1.

**Table 1:** Overview of the updated *TP53*-specific ACMG/AMP criteria (v2.3.0) developed by the ClinGen *TP53* VCEP*

Original ACMG/AMP guidelines			*TP53* specifications summary (v2.3.0)☨
Code	**Short Description**	**Brief description of v2 update**	
PVS1	Null variant in a gene where loss of function is a known mechanism of disease	Gene specific updates to flowchart. Predicted effects from SpliceAI for possible 1,2 splice site variants	Follow SVI-approved *TP53*-specific PVS1 flowchart (Fig. 1)
PS1	Same amino acid change as a previously established pathogenic variant	Allow LP variants to count for moderate weight. Incorporation of SpliceAI prediction ≤ 0.1 to rule out splicing with equal weight as RNA data	**PS1:** Can be applied to variants asserted as Pathogenic following the *TP53* VCEP’s specifications **PS1_Moderate:** Can be applied to variants asserted as Likely Pathogenic following the *TP53* VCEP’s specifications Must confirm there is no difference using RNA data or SpliceAI (< 0.2). If both PS1 and PM5 are met, apply the strongest weight possible for each rule code, not to exceed a combined strength of 4 points
PS2	De novo (proven or assumed) in a patient with disease and no family history	Change in points system to be equivalent with Bayes points system. Removal of PM6. Addition of SHH medulloblastoma and mosaics	Follow point structure outlined in manuscript and summarized in Table 2 (for probands with multiple cancers, use the most specific/highest weight cancer to determine point application for that proband) **PS2_Very Strong:** ≥ 8 points **PS2:** 4–7 points **PS2_Moderate:** 2–3 points **PS2_Supporting:** 1 point
PS3	Well-established *in* vitro or *in* vivo functional studies supporting of a damaging effect	Updated flowchart based on LR work after removing Giacomelli DNE. Added Kawaguchi tetramerization assay. Added possibilities for small deletions based on Kotler data only. Added Funk as a possibility for other assays	**PS3:** Non-functional on Kato data AND LOF by the majority of available assays **PS3_Moderate:** Partially functional on Kato data AND LOF by the majority of available assays. **PS3_Supporting:** Non-functional on Kato data AND no LOF by Giacomelli BUT abnormal on Kawaguchi data data. **PS3_Supporting** may also be applied to missense variants with no available Kato data and small deletions with available Kotler data where the majority of available assays demonstrate LOF. Eligible functional studies include Kato [32], Funk [21], Giacomelli [33], Kotler [38], Kawaguchi [42], as well as other assays considered to provide relevant functional information after expert interpretation, as previously reported [13] Do not apply PS3 at any weight for “missense” variants if PP3 is applied based on SpliceAI, or if there is any laboratory evidence of splicing aberration for the genetic variant being assessed, for which PVS1_Variable Weight (RNA) might be considered instead. Downgrade to PS3_Moderate if PVS1_Strong is applied. Do not apply PS3 at any strength if PVS1 is applied at full strength Refer to Fig. 3 and Additional file 3: Table S3 for more details on the application of functional codes PS3 and BS3
PS4	The prevalence of the variant in affected individuals is significantly increased compared with the prevalence in controls	Added HER2+. Added PS4_Very strong. Requirement that for PS4 to be applied, variants needs to meet PM2_supporting rather than not meeting BA1/BS1. Accounted for potential double counting/circularity in PS4 and PS2. Clarification that PS4 and PS2 cannot be used together. Removed constitutional mosaics	Follow the point structure outlined in the manuscript and summarized in Table 3 **PS4 Very Strong:** ≥ 8 points **PS4:** ≥ 4–7.5 points **PS4_Moderate:** 2–3.5 points **PS4_Supporting:** 1–1.5 points Points attributed to HER2 status must only be applied in unrelated individuals who underwent multigene panel testing. Do not apply this code for probands with de novo *TP53* variants, in which case PS2_Variable Weight should be applied instead. Variant must meet PM2_Supporting in order for PS4 to be applied at any strength
PM1	Located in a mutational hotspot and/or critical and well-established functional domain	Addition of PM1_Supporting for 2–9 occurrences. Removal that PM5 and PM1 cannot be applied together	**PM1**: Missense variants within the following codons 175, 245, 248, 249, 273, 282. This code weight can also be used for germline missense variants seen in https://cancerhotspots.org with ≥ 10 somatic occurrences for the same amino acid change **PM1_Supporting:** Missense variants seen in cancerhotspots.org with 2–9 somatic occurrences for the same amino acid change
PM2	Absent/rare from controls in an ethnically matched cohort population sample	Change to rarity instead of absence, defined as an order of magnitude under BS1. Update to gnomAD v4. Excluded subpops influenced by founder effects; guidance for avoiding CHIP contamination	**PM2_Supporting:** Allele frequency < 0.00003 (0.003%) across the most recent version of gnomAD. If multiple alleles are present within any genetic ancestry group, allele frequency in that group must be < 0.00004 (0.004%), ignoring subpopulations influenced by founder effects, such as Ashkenazi Jewish, Finnish, Amish, and Middle Eastern, as well as “Remaining” If the variant being assessed does not meet any population rule codes (PM2, BA1, BS1), curators should recalculate the total allele frequency based on the number of alleles over 0.35 to assess whether PM2 may be met after excluding the low VAF alleles which are likely to represent CH contamination in the database
PM3	For recessive disorders, detected *in trans* with a pathogenic variant	NA	Excluded (refer to Supplementary Table S1 in Fortuno et al., 2021 [13] for more details)
PM4	Protein length changes due to in-frame deletions/insertions in a nonrepeat region or stop-loss variants	NA	Excluded (refer to Supplementary Table S1 in Fortuno et al., 2021 [13] for more details)
PM5	Missense change at an amino acid residue where a different missense change determined to be pathogenic has been previously observed	Incorporation of SpliceAI prediction ≤ 0.1 to rule out splicing with equal weight as RNA data. Addition of PM5_Strong. Different rationale for different weights based on the confidence of the previously asserted variants. Incorporation that PS1 and PM5 cannot exceed 4 points if applied together. Removal that PM5 and PM1 cannot be applied together. SpliceAI cutoff updated to be more permissive for non-silent/intronic variants	**PM5_Strong:** Missense variant at an amino acid residue where ≥ 2 different missense variants previously determined to be pathogenic according to the *TP53* VCEP’s specifications have been seen before **PM5:** Missense variant at an amino acid residue where 1 different missense variant previously determined to be pathogenic according to the *TP53* VCEP’s specifications has been seen before **PM5_Supporting:** Missense variant at an amino acid residue where 1 different missense variant previously determined to be likely pathogenic according to the *TP53* VCEP’s specifications has been seen before. The previously seen likely pathogenic variant must have clinical data that demonstrates pathogenicity (i.e. PS2, PS4, PP1) in order for it to count towards PM5_Supporting code application The variant being evaluated must be equal or worse using Grantham than the known pathogenic. Splicing should be ruled out with either RNA data or SpliceAI (< 0.2). If both PS1 and PM5 are met, apply the strongest weight possible for each rule code, not to exceed a combined strength of 4 points
PM6	Assumed de novo, but without confirmation of maternity and paternity	Excluded	Excluded (use PS2 instead)
PP1	Co-segregation with disease in multiple affected family members	Clarification of the cancers that count as disease, which are the same as for PS2. Counting through obligate carriers allowed. Use caution language added. Requirement added that variant must not meet BA1/BS1	**PP1_Strong:** Cosegregation must be observed in ≥ 7 meioses across > 1 family **PP1_Moderate:** Cosegregation must be observed in 5–6 meioses in/across 1 or more families **PP1:** Cosegregation must be observed in 3–4 meioses in/across 1 or more families Meioses should be counted for individuals who both carry the variant and have an applicable cancer (see Table 2). Do not apply PP1 if variant meets BA1/BS1 criteria. See CSPEC for additional rules details and considerations
PP2	Missense variant in a gene that has a low rate of benign missense variation	NA	Excluded (refer to Supplementary Table S1 in Fortuno et al., 2021 [13] for more details)
PP3	Multiple lines of computational evidence support a deleterious effect on the gene or gene product	Addition of moderate benign code. Addition of single amino acid deletions (BayesDel only) and exonic/intronic based on SpliceAI. Addition that the splicing effect should always be considered with SpliceAI	**PP3_Moderate:** aGVGD Class C65 and BayesDel score ≥ 0.16 for missense variants **PP3:** aGVGD class C25-C55 and BayesDel score ≥ 0.16 for missense variants; BayesDel score ≥ 0.16 for single amino acid in-frame deletions; SpliceAI ≥ 0.2 for exonic variants (including silent variants and apparent “missense” variants or “single amino acid in-frame deletions” for which there is a predicted splice effect) and intronic splice variants (excluding ± 1,2 positions) All variants should be assessed to consider if there are splicing effects predicted. PP3 should not be used in combination with PVS1 Refer to Fig. 2 and Additional file 2: Table S2 for more details on the application of in silico codes PP3, BP4, and BP7
PP4	Patient phenotype or family history is highly specific for disease with a single genetic etiology	New code	**PP4_Moderate:** At least 2 independent observations of the variant with VAF 5–25% **PP4:** Observation of the variant with VAF 5–35% (i.e., once or multiple times with VAF > 25–35% and/or once with VAF 5–25%) This evidence code assumes a somatic origin of the *TP53* variant, and therefore cannot be applied in the same individual in combination with points from other phenotype-based criteria, such as PS4, PS2, and PP1. This code should not be applied from an observation of the low VAF *TP53* variant in a patient with blood cancer. Do not apply this code if the variant meets BA1 or BS1. Variant must have been detected on MGPT in order for this code to be applied
PP5	Reputable source reports variant as pathogenic	NA	Excluded (refer to Supplementary Table S1 in Fortuno et al., 2021 [13] for more details)
BA1	Allele frequency is above 5% in Exome Sequencing Project, 100 Genomes, or ExAC	Usage of gnomAD FAF	FAF ≥ 0.001 (0.1%) in the most recent version of gnomAD continental subpopulations of a single genetic ancestry group, excluding genetic ancestry groups influenced by founder effects, such as Ashkenazi Jewish, Finnish, Amish, and Middle Eastern, as well as “Remaining”. Sub-population must have ≥ 2000 alleles tested and a minimum of 2 alleles present
BS1	Allele frequency is greater than expected for disorder	Usage of gnomAD FAF	FAF ≥ 0.0003 (0.03%) but < 0.001 in the most recent version of gnomAD continental subpopulations of a single genetic ancestry group, excluding genetic ancestry groups influenced by founder effects, such as Ashkenazi Jewish, Finnish, Amish, and Middle Eastern, as well as “Remaining”. Sub-population must have a ≥ 2000 alleles tested and a minimum of 2 alleles present
BS2	Observed in a healthy adult	Addition of BS2_Moderate weight. Clarification that the females must be unrelated. Exclusion of sarcoma at any age based on LR work, as sarcoma > 60 was a predictor of TP53 variant pathogenicity based on personal history analysis. Caveat not to count variants with VAF < 35%	**BS2:** ≥ 8 unrelated females who have reached at least 60 years of age without cancer. These individuals all must have come from a single source (single lab, database, etc.). Cases cannot be counted across sources. Individuals with a diagnosis of sarcoma ≥ 61 years of age should not be counted towards the BS2 total **BS2_Moderate**: 4–7 unrelated females who have reached at least 60 years of age without cancer. These individuals all must have come from a single source (single lab, database, etc.). Cases cannot be counted across sources. Individuals with a diagnosis of sarcoma ≥ 61 years of age should not be counted towards the BS2 total **BS2_Supporting:** 2–3 unrelated females who have reached at least 60 years of age without cancer. These individuals all must have come from a single source (single lab, database, etc.). Cases cannot be counted across sources. Individuals with a diagnosis of sarcoma ≥ 61 years of age should not be counted towards the BS2 total Variants with VAF ≤ 35%, suggestive of somatic origin, should not be included in these counts. Females counted towards BS2 should be unrelated probands
BS3	Well-established *in* vitro or *in* vivo functional studies show no damaging effect on protein function	Kawaguchi tetramerization assay added. Added possibilities for small deletions based on Kotler data only. Added Funk as a possibility for other assays	**BS3:** Functional on Kato data AND no evidence of LOF by the majority of available assays **BS3_Supporting:** Partially functional on Kato data AND no LOF by ALL available assays. **BS3_Supporting** may also be applied to missense variants with no available Kato data and small deletions with available Kotler data with no LOF by the majority of available assays. Eligible functional studies include Kato [32], Funk [21], Giacomelli [33], Kotler [38], Kawaguchi [42], as well as other assays considered to provide relevant functional information after expert interpretation, as previously reported [13] Do not apply BS3 at any weight for “missense” variants if PP3 is applied based on SpliceAI, or if there is any laboratory evidence of splicing aberration for the genetic variant being assessed, for which PVS1_Variable Weight (RNA) might be considered instead Refer to Fig. 3 and Additional file 3: Table S3 for more details on the application of functional codes PS3 and BS3
BS4	Lack of segregation in affected members of a family	Language clarification made to be consistent with other VCEPs and to avoid potential double counting/conflation with BS2	Lack of segregation in affected family members (i.e. family members diagnosed with LFS-associated cancers as described in Table 2)
BP1	Missense variant in a gene where primarily truncating variants cause disease	NA	Excluded (refer to Supplementary Table S1 in Fortuno et al., 2021 [13] for more details)
BP2	Observed *in cis/trans* with a pathogenic variant	Excluded	Excluded given reports of *TP53* pathogenic variants observed in trans
BP3	In-frame indels in a repetitive region without a known function	NA	Excluded (refer to Supplementary Table S1 in Fortuno et al., 2021 [13] for more details)
BP4	Multiple lines of computational evidence suggest no impact on gene or gene product	Addition of moderate benign code. Addition of single amino acid deletions (BayesDel only) and silent/intronic outside splice sites based on SpliceAI. Addition that the splicing effect should always be considered with SpliceAI AND ruled out for BP4 at any weight for all variants. SpliceAI cutoff updated to be more permissive for non-silent/intronic variants	**BP4_Moderate:** BayesDel ≤ -0.008 irrespective of aGVGD score (except C65) AND no predicted differences in splicing (SpliceAI < 0.2) for missense variants **BP4:** BayesDel < 0.16 and > -0.008 irrespective of aGVGD score (except C65) AND no predicted differences in splicing (SpliceAI < 0.2) for missense variants; BayesDel score < 0.16 AND no predicted splicing impact (Splice AI < 0.2) for single amino acid in-frame deletions; SpliceAI ≤ 0.1 for silent or intronic variants (outside ± 1,2 positions) Refer to Fig. 2 and Additional file 2: Table S2 for more details on the application of in silico codes PP3, BP4, and BP7
BP5	Variant found in a case with an alternate molecular basis for disease	NA	Excluded (refer to Supplementary Table S1 in Fortuno et al., 2021 [13] for more details)
BP6	Reputable source reports variants as benign	NA	Excluded (refer to Supplementary Table S1 in Fortuno et al., 2021 [13] for more details)
BP7	Synonymous (silent) variant with no splicing impact AND the nucleotide is not conserved	Specification that BP4 needs to be met for BP7 to be met, and that BP7 can be applied not only to silent variants but also intronic. Specification of the location of both variant types to meet BP7: synonymous (silent) outside of the core splice motif (last three nucleotides and first nucleotide of the exon) or intronic variant at or beyond + 7 to − 21 positions. Addition of **BP7_Strong (RNA)** for silent or intronic variants with splicing assay data (regardless of location)	**BP7_Strong (RNA)**: A silent or intronic variant for which a well-established in vitro or in vivo functional study shows no damaging effect on protein function as measured by effect on mRNA transcript profile*,* as per recommendations in Walker et al., 2023 [18] **BP7:** A silent variant outside of the core splice motif (last three nucleotides and first nucleotide of the exon) or intronic variant at or beyond + 7 to -21 positions for which SpliceAI predicts no impact to the splice consensus nor the creation of a new splice site (BP4 is met, SpliceAI ≤ 0.1) Refer to Fig. 2 for more details on the application of in silico codes PP3, BP4, and BP7

*Abbreviations*: *CH* Clonal hematopoiesis, *CSPEC* ClinGen Criteria Specification Registry, *FAF* Filtering allele frequency, *MGPT* Multigene panel testing, *LFS* Li-Fraumeni syndrome, *LOF* Loss of function, *LP* Likely Pathogenic, *SVI* Sequence Variant Interpretation, *VAF* Variant allele frequency, *VCEP* Variant Curation Expert Panel

^*^To find the most current version of the *TP53* VCEP’s specifications, please visit the ClinGen Criteria Specification Registry at https://cspec.genome.network/cspec/ui/svi/svi/GN009

**☨**Refer to the manuscript Results or ClinGen Criteria Specification Registry for more information on code-specific caveats and requirements

### Population data (PM2, BA1, and BS1)

All population codes were updated to specify that the most recent version of gnomAD [27] should be used (gnomAD v4.1.0 at the time of these updates), allowing for earlier gnomAD versions or use of other databases if they are able to provide information the curator deems necessary for optimal accurate variant curation (e.g., more individuals of a certain subpopulation tested).

#### PM2—absence from controls or presence at low frequency

In anticipation of upcoming broader ACMG/AMP updates, the requirement for PM2_Supporting code application was updated from “absent from population databases” to “sufficient rarity” to better align with ClinGen SVI recommendations that will likely require the variant to be sufficiently rare for phenotype codes to apply. The VCEP established a PM2 cutoff that would identify variants with a population frequency consistent with being disease-causing by defining a threshold an order of magnitude under BS1 (i.e., PM2 allele frequency < 0.00003 (0.003%)), with no single genetic ancestry group having multiple alleles with a frequency higher than 0.00004 (0.004%). This second requirement does not include groups influenced by founder effects and/or bottleneck effect: Ashkenazi Jewish, Finnish, Amish, Middle Eastern, or the “Remaining” group.

To account for potential contamination of variants caused by clonal hematopoiesis (CH) in population databases, if the variant being assessed does not meet any population rule code, the curator should recalculate the total allele frequency based on the number of alleles with a variant allele fraction (VAF) over 0.35 to assess whether PM2_Supporting may be applied after excluding low VAF alleles that are likely to represent CH and/or sequencing technical issues (i.e., likely not germline). This can be done by visualizing the “allele balance” for heterozygotes under the genotype quality metrics for a given variant. By hovering over the histogram bars, the number of variant carriers for each bar between 0.35 and 0.65 can be totaled and this can be used to revise the allele count to determine the allele frequency that can be used to assess if PM2_Supporting can be met. This keeps the same genetic ancestry group frequency requirement, given that it is not possible to identify to which group low VAF alleles belong to.

#### BA1 and BS1—stand-alone and strong evidence for benign variation based on frequency of variant in a general population

Based on SVI recommendations to avoid variation in allele frequency estimation based on population size, the VCEP conservatively elected to use the filtering allele frequency (FAF), which represents the maximum credible genetic ancestry group allele frequency, instead of minor allele frequency for application of the benign population codes, maintaining the same previously established frequency thresholds (≥ 0.001 or 0.1% for BA1 and ≥ 0.0003 or 0.03% for BS1). Utilization of FAF is based on ClinGen guidance to VCEPs regarding the use of gnomAD v4 [28].

This includes a requirement of a minimum of 2000 alleles tested in a continental subpopulation of a single genetic ancestry group excluding those influenced by founder effects and is not to be calculated for singleton occurrences. Although manual review of variants meeting BA1 showed that low VAF generally accounts for < 5% of the total allele count, the VCEP added caution for variants meeting benign frequency codes where the majority of alleles are below 35% VAF.

### Computational and predictive data (PVS1, PS1, PM5, PP3, BP4, and BP7)

#### PVS1—very strong evidence of pathogenicity for null variants where loss of function is a known mechanism of disease

**Fig. 1:**
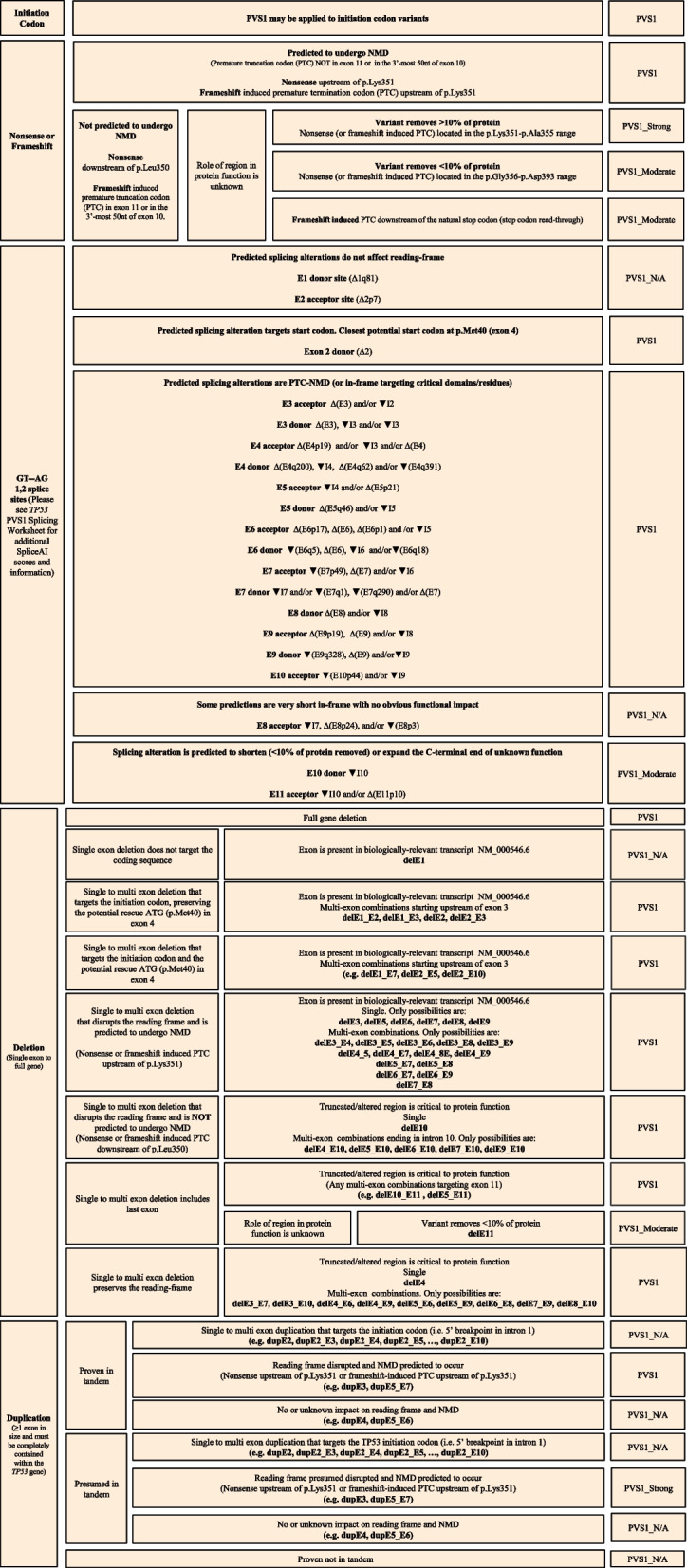
Flowchart for the application of PVS1 to *TP53* null variants in relation to the transcript NM_000546.6. Splicing predictions for GT-AG sites are based on SpliceAI and available experimental data. PVS1_Variable Weight (RNA) may be applicable for variants with RNA-based assay data demonstrating aberration (see Additional file 1: Table S1). Δ = exon skipping, ▼ = intron retention

The VCEP expanded on previously published ClinGen recommendations for PVS1 code application [29] to provide *TP53*-specific caveats and recommendations. These changes are summarized in an adapted flowchart detailing code weight application for *TP53* initiation codon, nonsense/frameshift, and canonical splice site variants, as well as deletions and duplications (Fig. 1). There are many alternative transcripts of *TP53* but the main and most abundant is the MANE select transcript NM_000546.6, which contains the entire transactivation domain (TAD) and the longest C-terminal domain [30]; therefore, the VCEP considered this as the reference transcript. All domains were considered to be pertinent for protein function, including the TAD1 domain located upstream of the closest potential in-frame start codon at codon 40 [31]. However, the clinical relevance of the C-terminal end downstream of codon 350 is not clear due to conflicting functional assay results within those residues [32, 33]. Regardless, given the absence of empirical data justifying the strength of evidence for missing regions, the VCEP followed general loss-of-function recommendations [29] for protein regions of unknown function downstream of codon 350: PVS1_Strong if the deletion removes > 10% of the protein, and PVS1_Moderate if the deletion removes < 10% of protein. Consistent with PM4, alterations expanding the C-terminal end (stop codon read-through) were considered PVS1_Moderate. The VCEP used splicing predictions based on SpliceAI [34] and MaxEntScan (MES) [35] for GT-AG sites to inform strengths for GT-AG 1,2 splice sites variants as well as available experimental data, including minigene assays, RT-PCR experiments performed in carriers, and MutSpliceDB information retrieved through ClinVar as well as information from the Intron Retention Associated Variants (IRAV) Database [36]. This information, along with the proposed PVS1_Variable Weight code for each possible *TP53* splice site variant, is detailed in Additional file 1: Table S1.

To differentiate experimental splicing data from predictions, the VCEP considered the incorporation of RNA-based assay data demonstrating splicing aberration as PVS1_Variable Weight (RNA), following the Walker et al. [18] recommendations.

#### PS1 and PM5—same amino acid change or position as a previously established pathogenic variant

To improve usability, these codes may now be applied at various strengths based on the classification of the previously established pathogenic variants, which can be asserted as P/LP by any submitter if using the *TP53* VCEP’s specifications. In this regard, the VCEP introduced the option of using SpliceAI prediction to rule out splicing aberration (SpliceAI < 0.2) for variants with no RNA-based data. An additional code weight of PM5_Strong for variants with two or more previously established P variants was also added.

To avoid potential double-counting of evidence with both these codes which rely on evidence from other variants, a specification was added that, when used in conjunction, PS1 and PM5 must not collectively exceed + 4.0 points.

#### PP3 and BP4—computational predictors

The VCEP initially tested BayesDel [37] threshold ranges as proposed by the SVI [17] using two independent reference sets: original reference sets to guide v1 criteria for PP3 and BP4 (247 assumed pathogenic and 71 assumed benign variants based on functional and/or clinical data) [24] plus sets based on Giacomelli functional data (608 loss-of-function (LOF) variants versus 1955 no LOF variants) [33]. However, the resulting equivalent strengths from our analyses did not align with those proposed for each range; therefore, the VCEP explored more BayesDel ranges than the single cut point used (0.16), employing the same original reference sets. To determine these ranges, the VCEP calculated the average of the scores of pathogenic variants (0.444) and benign variants (− 0.008), establishing a total of four ranges with all three scores. The performance based on the LR-equivalent strengths using these scores alone was additionally assessed when combined with different groupings of Align-GVGD classes [24]. These results supported the addition of a moderate benign code and a reduction in the number of variants with “No evidence” from 458 to 348 of all possible *TP53* missense variants. Additionally, for BP4 or BP4_Moderate to be applied to a missense variant, the VCEP added the requirement that there must not be a positive prediction of splicing aberration (i.e., SpliceAI < 0.2).

PP3 and BP4 may now be applied for single amino acid in-frame deletions based on BayesDel only, after computing all scores for every possible p53 single amino acid deletion. A spreadsheet of these scores is available for curators in the Cspec.

The VCEP integrated the use of SpliceAI spliceogenicity prediction alone to the application of PP3 (SpliceAI ≥ 0.2) and BP4 (SpliceAI ≤ 0.1) for exonic and intronic variants outside the canonical splice sites, and for synonymous and intronic variants outside the canonical splice sites, respectively.

**Fig. 2:**
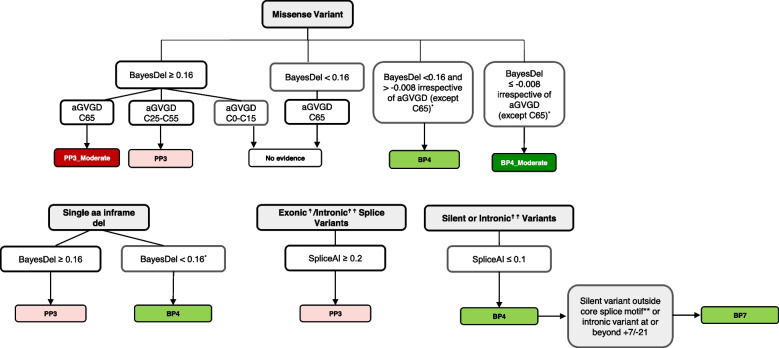
Flowchart for application of in silico codes PP3, BP4, and BP7 ^*^AND no predicted differences in splicing (SpliceAI < 0.2) ^☨^Including silent, and apparent “missense” or “single amino acid in-frame deletions” for which there is a predicted splice effect ^☨☨^Excluding ± 1,2 positions **Core splice motif includes last three nucleotides and first nucleotide of the exon

A flowchart simplifying the application of PP3 and BP4 rule codes is shown in Fig. 2. Pre-computed codes for every possible missense variant based on Align-GVGD and BayesDel, along with SpliceAI predictions, are available in the CSpec and also found in Additional file 2: Table S2.

#### BP7—synonymous (silent) variants with no splicing impact

The BP7 criterion, originally specified for synonymous (silent) variants only, was expanded to cover intronic variants and is applicable if the following criteria are met, consistent with other VCEPs and the Walker et al. [18] recommendations: (i) BP4 code is met, and (ii) silent variants are located outside of the core splice motif (last three nucleotides and first nucleotide of the exon) or intronic variants are located in the + 7 to − 21 region (both + 7 and − 21 included) (Fig. 2).

*BP7_Strong (RNA)* was incorporated as an alternative for silent or intronic variants with RNA-based assay data demonstrating no splicing aberration aligning with recommendations previously provided by Walker et al. [18].

### Functional data (PS3 and BS3)

#### PS3 and BS3—in vivo and in vitro functional studies

The VCEP continued to base the application of functional codes for missense and small in-frame events on systematic functional assays, incorporating those previously used [32, 33, 38], and allowing the inclusion of other assays to complement these results as justified in the original specifications [13]. The study by Kato et al. [32] remained as the primary assay due to its higher predictive performance in comparison to Giacomelli et al. [33], as previously explained [13]. Both these assays have results available for every possible missense variant, as opposed to Kotler et al. [38] and the recently published CRISPR-based published assay of Funk et al. [21], for which results are only available for a limited number of exons. We note that the flexibility to include additional relevant assays in the functional assay flowchart has allowed us to include the CRISPR-based assay [21] as a minor update to the major update to the specifications v2.

**Fig. 3:**
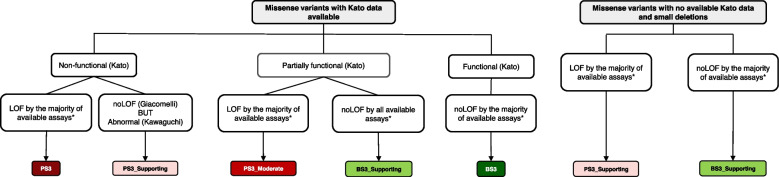
Flowchart for application of functional codes PS3 and BS3 via protein function for missense variants (left) and small deletions (right) *Eligible functional studies include Kato [32], Funk [21], Giacomelli [33], Kotler [38], and Kawaguchi [42], as well as other assays considered to provide relevant functional information after expert interpretation, as previously reported [13]

Following on previous work suggesting that the dominant-negative effect (DNE) category used in Giacomelli et al. [33] did not add substantial predictive accuracy to the LOF category [39], DNE was excluded following quantitative reanalysis of original reference sets used for PS3 and BS3 calibration (52 assumed pathogenic variants found in LFS patients versus 31 assumed benign variants found in non-cancer controls) [13]. To better understand the functional consequences of variations in the p53 tetramerization domain, the VCEP reviewed studies performing tetramerization assays [40–48] and selected the largest study by Kawaguchi et al. [42] for inclusion in the functional codes, after comparison of the results against existing functional results assisted by expert judgment, adding a new PS3_Supporting category. Additional modifications included adding PS3_Supporting and BS3_Supporting categories to accommodate variants with no yeast-based transactivation data, including small deletions. The choice of a supporting strength was conservatively made due to insufficient clinical data available to effectively evaluate these variants. The updated functional flowchart can be seen in Fig. 3. Pre-assigned codes based on these assays are available in the Cspec and also found in Additional file 3: Table S3.

Considering that the functional assays of variants utilized in the PS3/BS3 flowchart were done at the protein level, caveat language was introduced preventing the application of these codes if there is aberrant splicing predicted by SpliceAI (SpliceAI ≥ 0.2). The same applies if there is any RNA-based assay evidence of splicing aberration, for which PVS1_Variable Weight (RNA) should be considered instead [18].

### Hotspot data (PM1)

#### PM1—variants in critical gene regions or hotspots

The VCEP expanded this criterion by introducing a supporting code weight (PM1_Supporting) for variants with two to nine occurrences in the Cancer Hotspots Database [49], following the recommendations from the ClinGen Germline/Somatic Variant Subcommittee [50]. Since this criterion mostly applies to specific missense changes based on their tumor frequency, the previous limitation that PM5 and PM1 cannot be applied together was removed.

### Clinical data (PS2, PS4, PM6, PP1, PP4, BS2, and BS4)

#### *De novo* data (PS2 and PM6)

##### PS2 and PM6—De novo occurrences

The *TP53* VCEP elected to adopt PS2 as the sole de novo code and to exclude PM6 as redundant to improve the usability of codes. The v1 point system was modified to align with the Bayesian point system [16], allowing for 1, 2, or 4 points towards a de novo score based on the phenotype of the proband and whether parental relationships are confirmed (e.g., trio genome, exome or maternity/paternity testing) or unconfirmed (Table 2). The VCEP clarified that confirmed de novo probands include those with confirmed constitutional somatic mosaicism (low *TP53* VAF on blood or buccal testing with the variant detected in non-lymphocyte tissue and/or segregating in children). The VCEP also addressed a discrepancy related to the assigned strengths for hypodiploid acute lymphoblastic leukemia and sonic hedgehog-activated medulloblastoma between the published manuscript [13] and the two versions of the *TP53* specifications (v1 and v1.2) accessible on the ClinGen *TP53* VCEP website, leaving both in at moderate strength; breast cancer age cut-offs for strong and moderate strengths were also clarified. While it is specifically low hypodiploid acute lymphoblastic leukemia (ALL) (32–39 chromosomes) that is associated with germline *TP53* variants, the VCEP noted that this granular level of information is not always available to individuals curating *TP53* variants. We therefore were deliberate in omitting this specification from the PS2/PP1 Table 2 to allow for the application of these codes in cases where the curator felt counting a hypodiploid ALL case towards PS2 or PP1 codes was appropriate even if they did not have details on chromosome count or if the case was not specifically labeled “low hypodiploid.” De novo occurrences counted towards PS2 should not be counted towards PS4 code application to avoid double counting phenotypic presentation as evidence towards pathogenicity.

**Table 2:** LFS Cancers and points for applying PS2 and PP1 codes

Points per proband		Proband cancer	
**Strongly associated LFS cancers**			
● 4 points for probands with a strongly associated cancer when maternity and paternity are confirmed ● 2 points for probands with a strongly associated cancer when maternity and paternity are assumed		● Breast cancer (including ductal carcinoma in situ) < 31 years of age ● Choroid plexus carcinoma ● Adrenocortical adenoma or carcinoma < 18 years of age ● Rhabdomyosarcoma or osteosarcoma < 46 years of age	
**Moderately associated LFS cancers**			
● 2 points for probands with a moderately associated cancer when maternity and paternity are confirmed ● 1 point for probands with a moderately associated cancer when maternity and paternity are assumed		● Breast cancer (including ductal carcinoma in situ) ≥ 31 and < 50 years of age ● Malignant brain tumor (excluding optic glioma) < 46 years of age ● Adrenocortical adenoma or carcinoma ≥ 18 and < 50 years of age ● Primary lung cancer (excluding carcinoid tumors) < 46 years of age ● Rhabdomyosarcoma or osteosarcoma > 45 years of age ● Other sarcoma (e.g., malignant breast phyllodes tumor, leiomyosarcoma, liposarcoma, etc.) < 60 years of age ● EXCEPT FOR dermatofibrosarcoma or Ewing sarcoma ● Hypodiploid acute lymphoblastic leukemia ● Sonic hedgehog-activated medulloblastoma	
**PS2 Code Strength Based on Total Points**			
**PS2_Supporting**	**PS2_Moderate**	**PS2**	**PS2_Very Strong**
1	2–3	4–7	≥ 8

#### Phenotype data (PS4, PP4, and BS2)

##### PS4—Variant-level case–control studies or proband counting

Application of + 1.0 point for probands or families meeting Classic LFS criteria and + 0.5 point for probands or families meeting Revised Chompret criteria remained the same as in the v1 specifications. However, a caveat was added that this code should not be applied for patients with de novo variants, in which case PS2_Variable Weight should be applied instead, as mentioned above. Tumor pathology status, particularly HER2 + breast cancer, was added to this code, following findings from a study comparing data between *TP53* carriers and non-carriers from Ambry Genetics (*N* = 4240) and GeneDx (*N* = 23,244) [19]. According to this study, the LR towards pathogenicity was significant for women diagnosed < 40 years of age with a HER2 + breast tumor and equivalent to the supporting strength, and suggested a requirement of having two HER2 + *TP53* carriers, which avoids putting too much emphasis on a single data point. Therefore, individuals with HER2 + breast cancer under the age of 40 years may also be given + 0.5 point based on Fortuno et al. findings [19], with the caveat that these individuals must have undergone multi-gene panel testing (MGPT), in agreement with the data used to calibrate this new evidence type. As in v1, points are tallied to determine the PS4 code strength application. For specific details on the updated point systems for the specifications of the PS4 criterion, refer to Table 3.

**Table 3:** Evidence types and points for PS4 code application

Evidence type	Single observation		Points per proband
Clinical criteria	Revised Chompret Criteria		0.5
	Classic LFS Criteria		1
Tumor pathology	HER2 + breast cancer < 40 years		0.5
**PS4 Code Strength Based on Total Points**			
**PS4_Supporting**	**PS4_Moderate**	**PS4**	**PS4_Very Strong**
1–1.5	2–3.5	4–7.5	≥ 8

Finally, in anticipation of the upcoming major ACMG guidelines updates, more recent SVI recommendations to VCEPs require that the variant is “rare” (i.e., meet the VCEP threshold for PM2_Supporting) to consider patient phenotype for case counting criteria (PS4 for dominantly inherited monogenic disease entities and PM3 for recessively inherited monogenic disease entities). The *TP53* VCEP’s v2 specifications therefore require PM2_Supporting to be met to apply PS4 at any strength.

##### PP4—Patient phenotype highly specific for disease

Utilizing recent data demonstrating that *TP53* variants observed at low VAF are significantly more likely to be pathogenic than benign on the basis of being suspected drivers of clonal hematopoiesis (CH) [20], the VCEP incorporated this evidence type into our updated specifications under a criterion that our VCEP had previously excluded for this gene, PP4. In the referenced study, which draws data from two independent diagnostic laboratories (Peter MacCallum Cancer Centre and Ambry Genetics), the observation of a *TP53* variant in blood with a VAF between 25 and 35% had an LR equivalent to the moderate strength, and a VAF between 5 and 25% had an LR equivalent to the strong strength. To minimize the chance of overestimating the strength of the evidence, the VCEP adjusted this criterion by downgrading each VAF range, assigning them to PP4 and PP4_Moderate, respectively, with the latter requiring at least two observations. Since this criterion assumes a somatic origin of the *TP53* variant being classified, it cannot be applied in the same individual with points from other phenotype-based criteria, such as PS4, PS2, and PP1. It also cannot be applied from an observation of the low VAF variant in a patient with blood cancer, given that the variant would likely be a consequence of this. Further, the variant must have been detected in MGPT for this code to be applied, in agreement with the original datasets in which this criterion was calibrated, and not when the indication for testing is presentation with an LFS cancer. In patients with LFS features and a *TP53* variant at a low VAF, postzygotic somatic mosaicism is a more likely mechanism than CH (see PS4). This code may not be applied if the variant also meets either BA1 or BS1 criteria.

##### BS2—Observed in a healthy individual

Clarifying language was added to specify that individuals contributing to this criterion must be “unrelated” females, based on the underlying data calibrating the definition of each strength. A moderate strength for four to seven individuals based on the corresponding LR was added. Finally, sarcoma occurring in individuals older than 60 years was excluded as a phenotype that contributes to this criterion based on unpublished internal findings from personal history analyses in MGPT data in which sarcoma at any age was a significant predictor of *TP53* variant pathogenicity. A caveat was added preventing the inclusion of individuals with a variant with a VAF at or below 35%, which is suggestive that the variant may be of somatic origin.

#### Segregation data (PP1 and BS4)

##### PP1—Co-segregation with disease

To ensure consistency in the application of this criterion, the VCEP limited the cancers considered as “disease” to the same cancers considered informative for the PS2 criterion (Table 2). The VCEP also clarified that co-segregations may be counted through obligate unaffected heterozygotes as well as families with only tumor testing available where the variant has been demonstrated in the germline of at least one individual in the family. In these cases, if the VAF in the tumor is not consistent with the variant being heterozygous, it should not be counted towards co-segregations. Caveat language was added that this code may not be applied if the variant also meets BA1 or BS1 criteria. The VCEP urges caution in counting co-segregations in families where breast cancer is the only malignancy. It is preferable that other breast cancer predisposition syndromes have been ruled out by genetic testing, but this is not required to count co-segregations.

##### BS4—Lack of segregation with disease

In order to avoid potential risk of double-counting with BS2, BS4 may only be applied if a proband has a phenotype-positive (with LFS-associated cancers as per Table 2) genotype-negative first-, second-, or third-degree relative. Living, unaffected individuals within a family harboring a given variant cannot be used as evidence against variant pathogenicity under this particular criterion.

### *Cis/trans* testing data (BP2)

#### BP2—Observed in *cis/trans*

While initially included in the v1 specifications, the *TP53* VCEP elected to remove this code from the updated specifications given reports of multiple *TP53* pathogenic variants observed in *trans*, particularly for the Brazilian founder variant NM_000546.6(TP53):c.1010G > A (p.Arg337His) [51–53].

### Update to a modified Bayesian point system for evidence code combinations and final variant classification

To provide curation simplicity and the ability to more empirically calibrate different evidence types, and in alignment with practices among other VCEPs and upcoming anticipated ACMG/AMP updates, the VCEP adopted the Bayesian point system as proposed by Tavtigian et al. [16], building on the previous Bayesian reanalysis of the ACMG/AMP guidelines [54].

In this Bayesian point system, each evidence code is assigned a positive or negative value proportional to its level of evidence. For each variant being assessed, the points corresponding to all met criteria are summed, leading to a final point value. This final point value is then used to determine the variant classification based on the point totals specified in Table 4. The VCEP changed the point threshold to reach the LB classification from − 1.0 to − 2.0, in agreement with the original ACMG/AMP guidelines, except for variants with at least two benign evidence codes where PM2_Supporting is the only pathogenic evidence code applied.

**Table 4:** Equivalencies between ACMG/AMP levels of strength and equivalent points, and point ranges to reach each variant class, as per Tavtigian et al., 2020 [16]

Code strength	Supporting	Moderate	Strong	Very strong
Pathogenic points	+ 1	+ 2	+ 4	+ 8
Benign points	− 1	− 2	− 4	− 8
**Category**		**Point Ranges**		
Pathogenic		≥ 10		
Likely Pathogenic		6 to 9		
Uncertain		0 to 5		
Uncertain^a^		− 1		
Likely Benign		− 2 to − 6		
Benign		≤ − 7		

^a^A final point value of − 1 may be overridden to Likely Benign in cases where at least two benign evidence codes are applied

### Pilot variant reclassifications using updated specifications

**Fig. 4:**
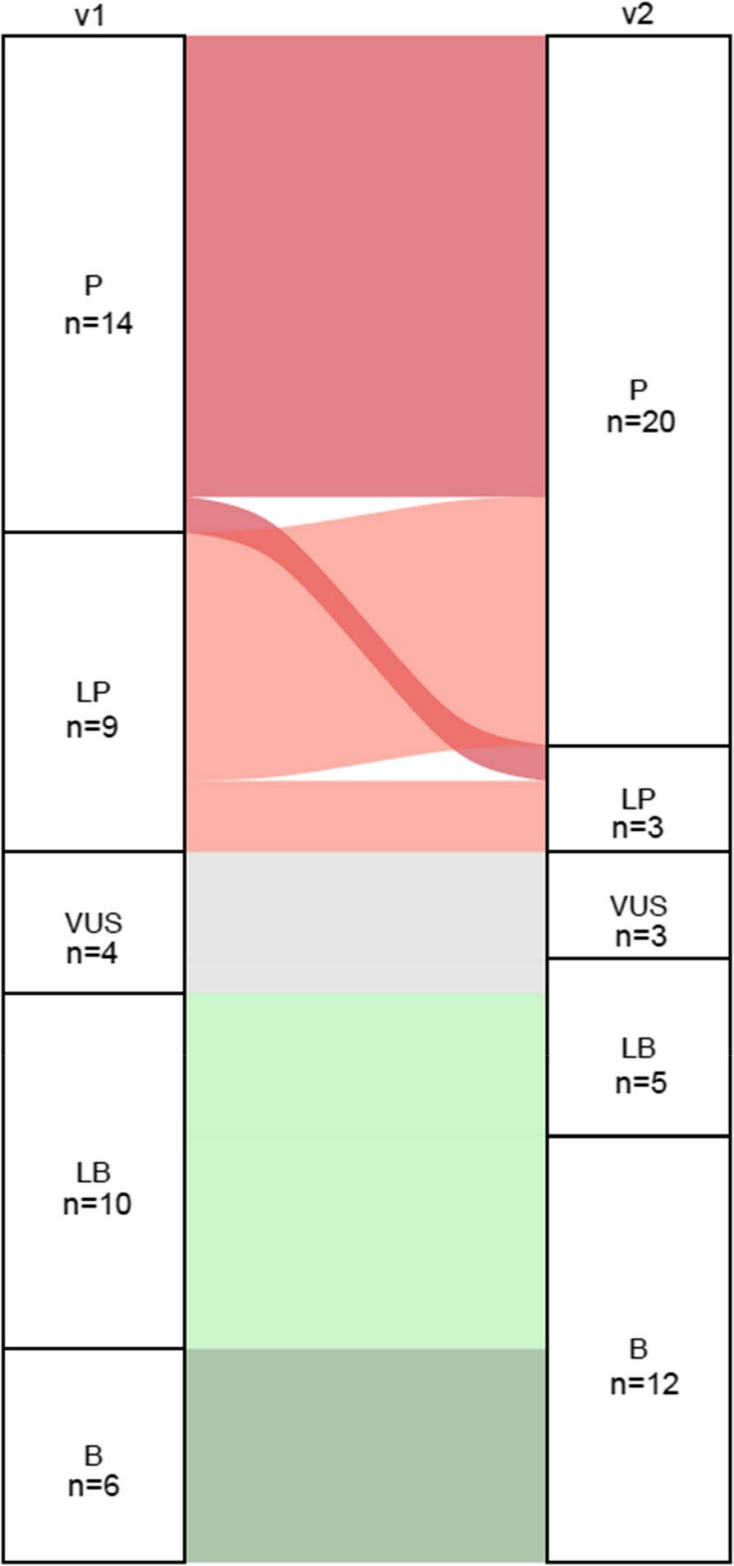
Summary of results for the 43 *TP53* pilot variants undergoing reclassification using the updated *TP53* VCEP’s specifications (v2) in comparison to the v1 VCEP specifications (v1). Abbreviations: B = Benign, LB = Likely Benign; LP = Likely Pathogenic P = Pathogenic; VUS = Variant

The updated specifications were piloted on the original 43 v1 pilot variants. Using the updated specifications and modified Bayesian point system, clinically meaningful classifications were achieved for 93% (40/43) of the pilot variants. Specifically, 78% (7/9) of previously LP variants were upgraded to P, and 60% (6/10) of LB variants were downgraded to B. Of the four VUS, one was resolved to LB and three remained VUS, including a suspected low penetrance Ashkenazi Jewish founder variant (NM_000546.6(TP53): c.1000G > C (p.Gly334Arg) [55]. The classifications achieved in comparison to previous specifications are summarized in Fig. 4.

## Discussion

This report describes the ongoing efforts of the ClinGen *TP53* VCEP to enhance *TP53* germline variant classification. Between 2019 and 2024, the *TP53* VCEP has developed and revised specifications that incorporate general recommendations for the use of ACMG/AMP criteria which were developed by the ClinGen SVI. By doing so, the VCEP successfully addressed some of the key challenges in classifying *TP53* germline variants that the VCEP encountered when curating variants using the original specifications, as well as challenges to *TP53* curation that were highlighted by VCEP members who are experts in the field. Since SVI approval of the v2 specifications in July of 2024, the VCEP has re-curated 70 variants that were due for updates per the ClinGen VCEP Standard Operating Protocol requirements for reanalysis [56] and has begun curation of newly recognized, never-before curated variants as of March 2025. The VCEP has curated and deposited in ClinVar 125 *TP53* classified variants to date.

These v2 specifications utilize MONDO ID (MONDO:0018875), which is equivalent to the ClinGen monogenic disease entity *TP53*-related Li-Fraumeni syndrome. Initially, the MONDO IDs for genes associated with LFS included LFS1 (*TP53*) and LFS2 (*CHEK2*). In a single early report, *CHEK2* was associated with a family with “Li-Fraumeni-like syndrome;” however, this was refuted by multiple subsequent reports [1]. Nevertheless, this association persisted in publications and widely used public resources such as MONDO [1]. MONDO has since retired the LFS2 designation, clarifying the sole association of *TP53* with the disease entity of LFS.

The results from the pilot demonstrate that the updated specifications result in strengthened variant classifications including a decrease in VUS classifications which may lead to improved clinical care for individuals with *TP53* germline variants. Likely Benign variants that were strengthened to benign did so primarily because of the incorporation of a moderate weight for BP4 along with updated clinical data leading to the application of BS2. The addition of a BP4_Moderate code also allowed for the clarification of one previous VUS variant to Likely Benign. Likely Pathogenic variants became more certain in large part due to the strengthening of phenotype codes PP4 and PS4 allowing for the application of phenotype data in scenarios where it previously could not be applied (e.g., HER2 + status, VAF data) along with the inclusion of a PP1_Moderate code and extensive updating of the PVS1 code to reflect the specifics of the *TP53* gene. The incorporation of new evidence types, updated computational, functional, and phenotype data utilizing quantitative likelihood ratio-based analysis, and a naturally scaled Bayesian point system for final variant classification, has improved the accuracy of *TP53* variant classification. A major impact of any VCEP specification is their public availability, facilitating the application of updated curation guidelines by individual diagnostic labs, and a reduction in conflicts and VUS through broad availability and consistent application of available evidence.

While *TP53* germline variants are generally rare and lack of clinical data can complicate classification, this gene has a rich availability of functional data in comparison to other genes which presents an advantage for curation. Several multiplexed assays of variant effect (MAVEs) have been performed for *TP53* which allows for the assessment of thousands of variants simultaneously, including all possible *TP53* missense variants [21, 32, 33, 38]. These assays have been included as part of the current *TP53* functional criteria specifications. The *TP53* VCEP is the first VCEP to use low VAF variants identified in blood samples as evidence towards pathogenicity to inform PP4 code application. Published work by a subset of VCEP members, demonstrating that low VAF positively correlates with variants undergoing positive selection as drivers of CH [20] allowed the VCEP to revise and incorporate an additional evidence type not included in the original ACMG/AMP guidelines. This update allows application of a pertinent phenotype code that was previously missing in this scenario given that *TP53* is included in most MGPTs and is increasingly being found as a secondary finding among patients that do not have LFS indication for testing [57]. Importantly, the VCEP generally follows a robust data-driven approach to inform the applicability and weight of most rule codes to ensure that the specifications are evidence-based, enhancing the validity of variant classifications.

Despite the described improvements, these current specifications have several limitations. First, the updated *TP53*-related Li-Fraumeni syndrome specifications were developed to distinguish highly penetrant variants associated with the classic LFS phenotype clinical criteria and thus lack sensitivity to identify reduced penetrance variants or those associated with less typical LFS phenotypes. The LFS phenotype has specific, well-defined clinical criteria known to be associated only with the *TP53* gene. The *TP53*-related Li-Fraumeni syndrome VCEP specifications were developed for the phenotypic entity of LFS, specifically, and not the broader *TP53*-related cancer predisposition spectrum that is increasingly recognized by the field [5]. However, individuals with atypical LFS phenotypes can also harbor *TP53* pathogenic germline variants. Further investigation of clinical and functional characteristics associated with *TP53* reduced penetrance germline variants and the spectrum of potential clinical manifestations is warranted to improve and/or develop more appropriate specifications. Following this, the phenotype currently used for PS4 may be considered limited, even for highly penetrant alleles, as probands/families are required to meet relatively strict clinical criteria. This might result in variants identified in probands with LFS-associated cancers outside the core spectrum missing on points towards this criterion. For this reason, a binary logistic regression-based approach considering independent significant phenotypes may be considered in future updates [58]. In this regard, given that the issue of reduced penetrance extends beyond *TP53*, expected guidance from the ClinGen Low Penetrance/Risk Allele working group will be valuable to ensure that any extension of our *TP53*-related Li-Fraumeni syndrome specifications is broadly consistent with general recommendations for tackling reduced penetrance variants. Similarly, the segregation and de novo evidence used by these specifications also rely on a limited number of selected cancers, in addition to assuming a high penetrance. Second, despite the availability of several *TP53* MAVEs, only one of these includes a “partial function” category, and most non-missense variants such as single amino acid deletions outside the DNA-binding domain or insertions lack any functional data. Finally, while the updated specifications do include several somatic features, namely HER2 + breast tumor status, VAF, and frequency in tumors, there could be a benefit in considering other somatic events in tumors that might be indicators of germline variant pathogenicity [59, 60].

The ClinGen *TP53* VCEP will continue to update the specifications as new evidence and recommendations emerge, for example structural-based bioinformatic tools such as AlphaMissense [61, 62]. The VCEP has resumed sustained ongoing curation and will continue to deposit three-star expert panel reviewed, FDA-recognized variant classification to ClinVar for public use. Per ClinGen policy, medically significant differences (i.e., P/LP vs. VUS/LB/B) are recommended to be reviewed within 6 months, and VUS vs. LB/B confidence differences as well as all VUS and LP variants are recommended to be reviewed every 2 years. LB variants are required to be updated whenever a major population database update is available, which has recently occurred with the gnomAD v4 release [27]. While this paper refers to the v2 of the *TP53*-related Li-Fraumeni syndrome specifications, a major update in comparison to the previous version, we anticipate periodic revisions that may not be simultaneously accompanied by a publication.

## Conclusions

The updated *TP53*-related Li-Fraumeni syndrome specifications developed by the ClinGen *TP53* VCEP have improved variant classification for germline *TP53* variants, strengthening the classification of over a third of pilot variants and overall achieving clinically meaningful classifications for 93% of curated pilot variants. The revised criteria incorporate the novel evidence type of clonal hematopoiesis creating a model for other VCEPs. As most of the criteria have been assessed and weighted using quantitative likelihood ratio-based analysis, this will enable a smoother transition to anticipated broad ACMG/AMP v.4.0 standards that will incorporate a more systematic Bayesian approach. Utilization of the *TP53*-related Li-Fraumeni syndrome specifications is expected to reduce the number of VUS and increase concordance between variant classifications, thus improving medical management for individuals with germline *TP53* variants. To find the most current version of the *TP53*-related Li-Fraumeni syndrome VCEP’s specifications, please visit the ClinGen Cspec [63].

## Supplementary Information

Additional file 1: Table S1. SpliceAI predictions and experimental splice data used to inform strengths for GT-AG +/-1,2 dinucleotide splice site variants in the *TP53* VCEP Specifications v2 (.xls).

Additional file 2: Table S2. Bioinformatic predictions and corresponding PP3 and BP4 codes for every possible p53 missense variant using the *TP53* specifications v2 (.xls).

Additional file 3: Table S3. Functional results for selected assays and corresponding preliminary PS3 and BS3 codes for every possible p53 missense variant using the *TP53* specifications v2 (.xls).

## Data Availability

All datasets used to calibrate and test these specifications are publicly available in the literature or databases. The updated specifications described in this study (version 2.3.0) are available at the ClinGen Cspec [63]. ClinGen TP53 VCEP variant assertions are publicly available in both the ClinVar database [14] and the ERepo [15].

## Abbreviations

ACMG/AMP: American College of Medical Genetics and Genomics and the Association for Molecular Pathology
ALL: Acute lymphoblastic leukemia
B: Benign
CH: Clonal hematopoiesis
ClinGen: Clinical Genome
Cspec: Criteria Specification Registry
FAF: Filtering allele frequency
FDA: Food and Drug Administration
MGPT: Multigene panel testing
LB: Likely Benign
LFS: Li-Fraumeni syndrome
LP: Likely Pathogenic
LR: Likelihood ratio
MAVE: Multiplexed assays of variant effect
P: Pathogenic
SVI: Sequence Variant Interpretation
VAF: Variant allele fraction
VCEP: Variant Curation Expert Panel
VUS: Variant of uncertain significance
